# Supplementary material for: High-Throughput Chlorophyll and Carotenoid Profiling Reveals Positive Associations with Sugar and Apocarotenoid Volatile Content in Fruits of Tomato Varieties in Modern and Wild Accessions
Source: Metabolites. 2021 Jun 18;11(6):398. doi: 10.3390/metabo11060398 (PMC8233878; doi:10.3390/metabo11060398)
Supplement: Supplementary file 1 [file metabolites-11-00398-s001.zip › supplementary_figures.pdf]

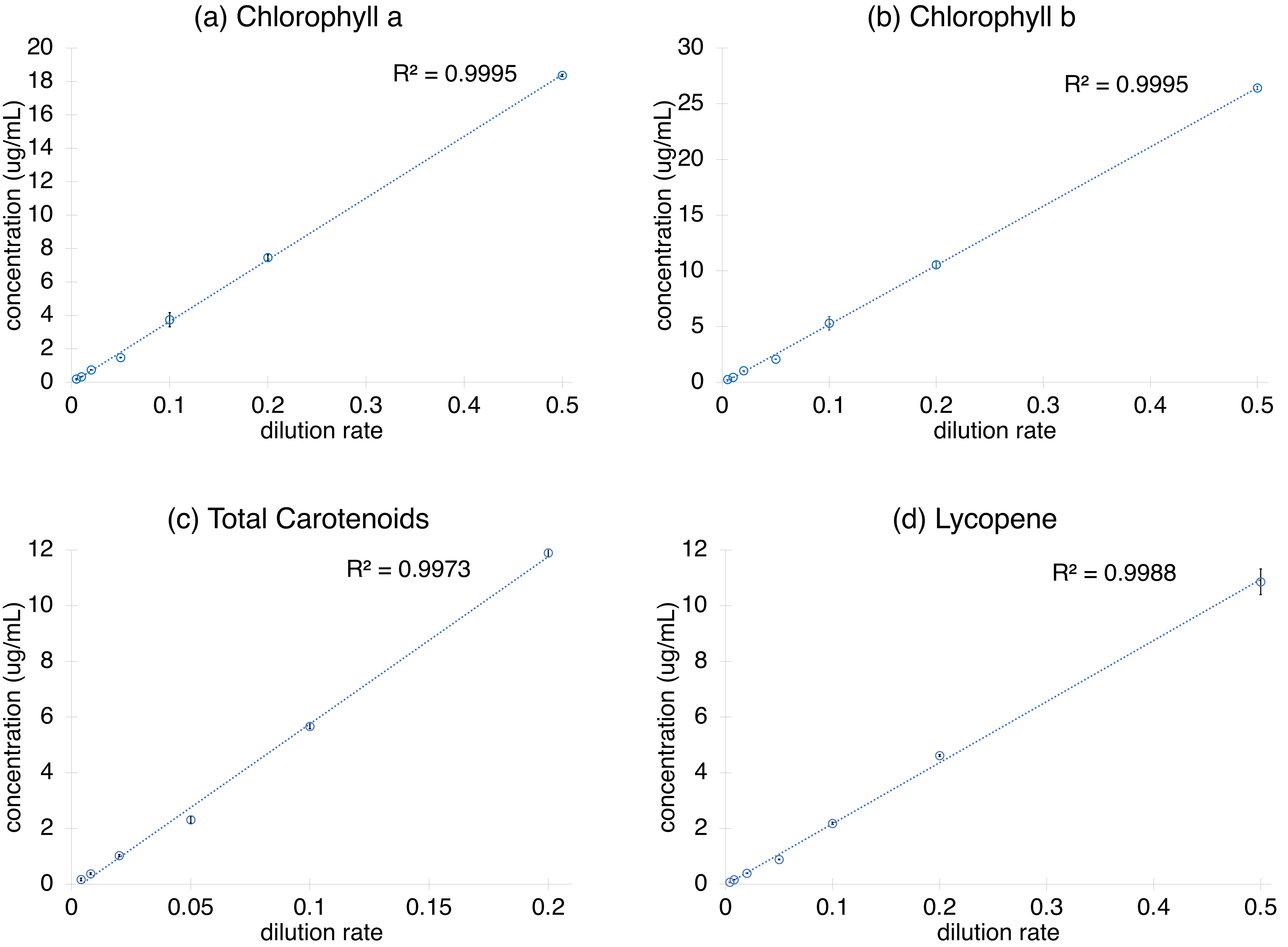

Figure S1. Calibration curve of dilution series. (**a**) Chlorophyll a, (**b**) chlorophyll b, (**c**) total carotenoids (sum of lycopene, *beta*-carotene, and lutein) and (**d**) lycopene standards were dissolved with acetone and quantified. Each value is shown as the average of analytical replicates ($N = 3$) with standard deviation.

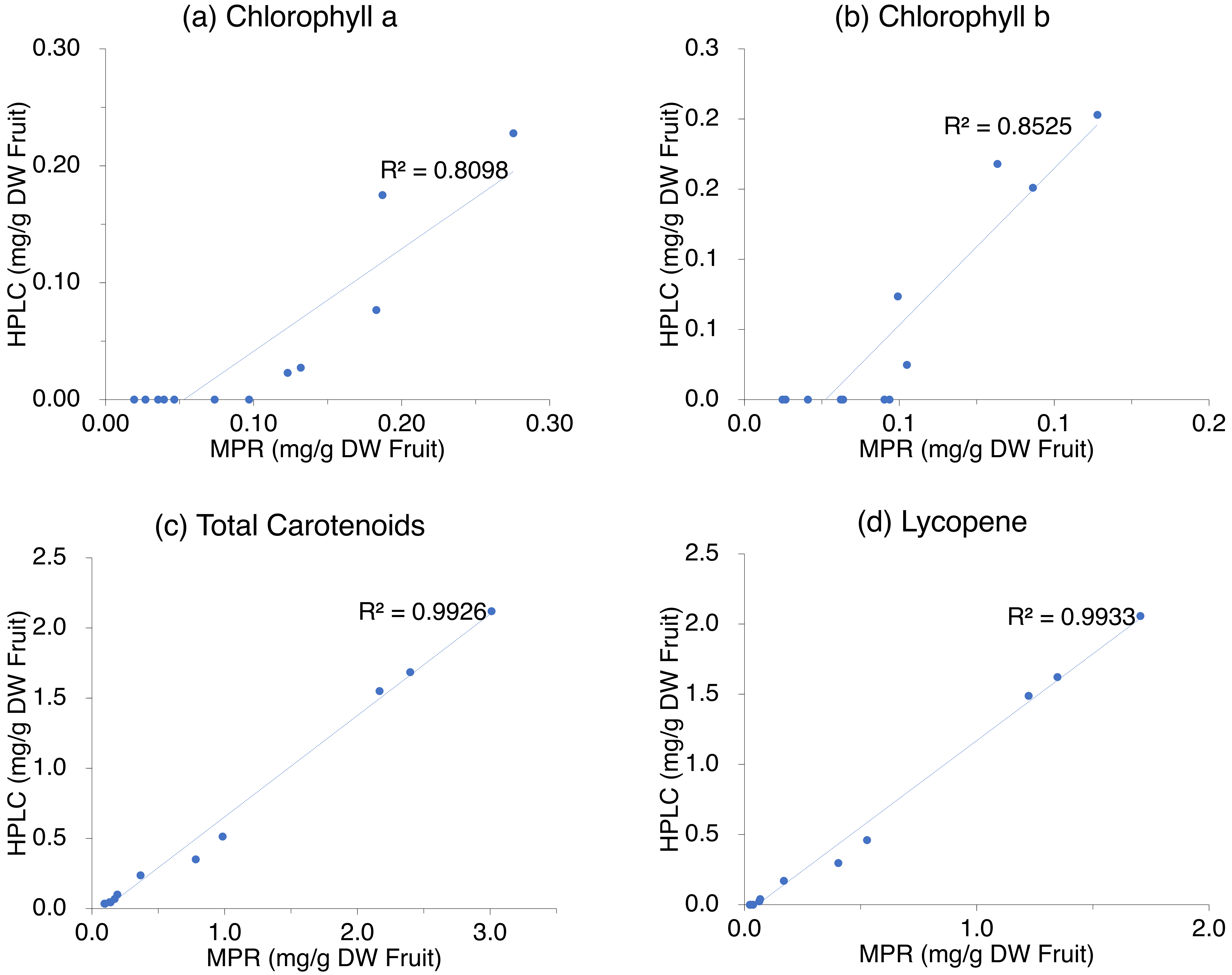

Figure S2. Plots of HPLC vs. microplatereader quantification results. (**a**) Chlorophyll a, (**b**) chlorophyll b, (**c**) total carotenoids and (**d**) lycopene content in same fruits were quantified by HPLC and MPR method (*n* = 12). The quantitative value of HPLC in (**c**) represents the sum of lycopene, *beta*-carotene and lutein.

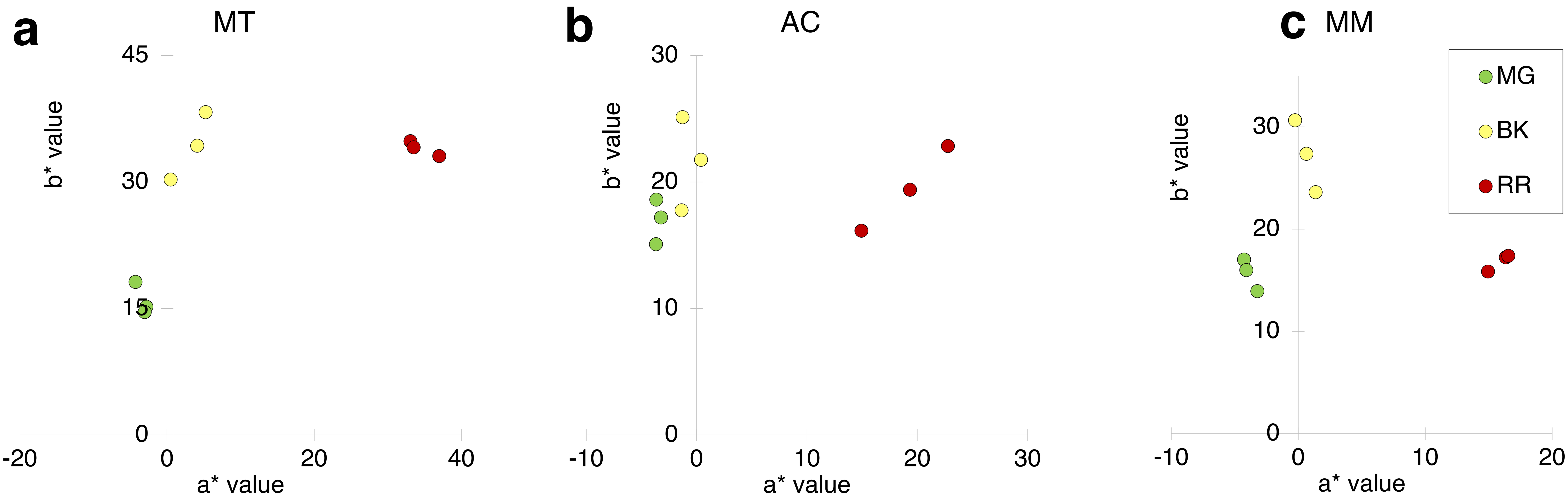

Figure S3. Color changes in fruits during ripening. (**a**) ‘Micro-Tom’ (MT), (**b**) ’Ailsa Craig’ (AC) ,and (**c**) ‘Money Maker’ (MM) measured by colorimeter (CM-700d, Konica Minolta, Tokyo, Japan).

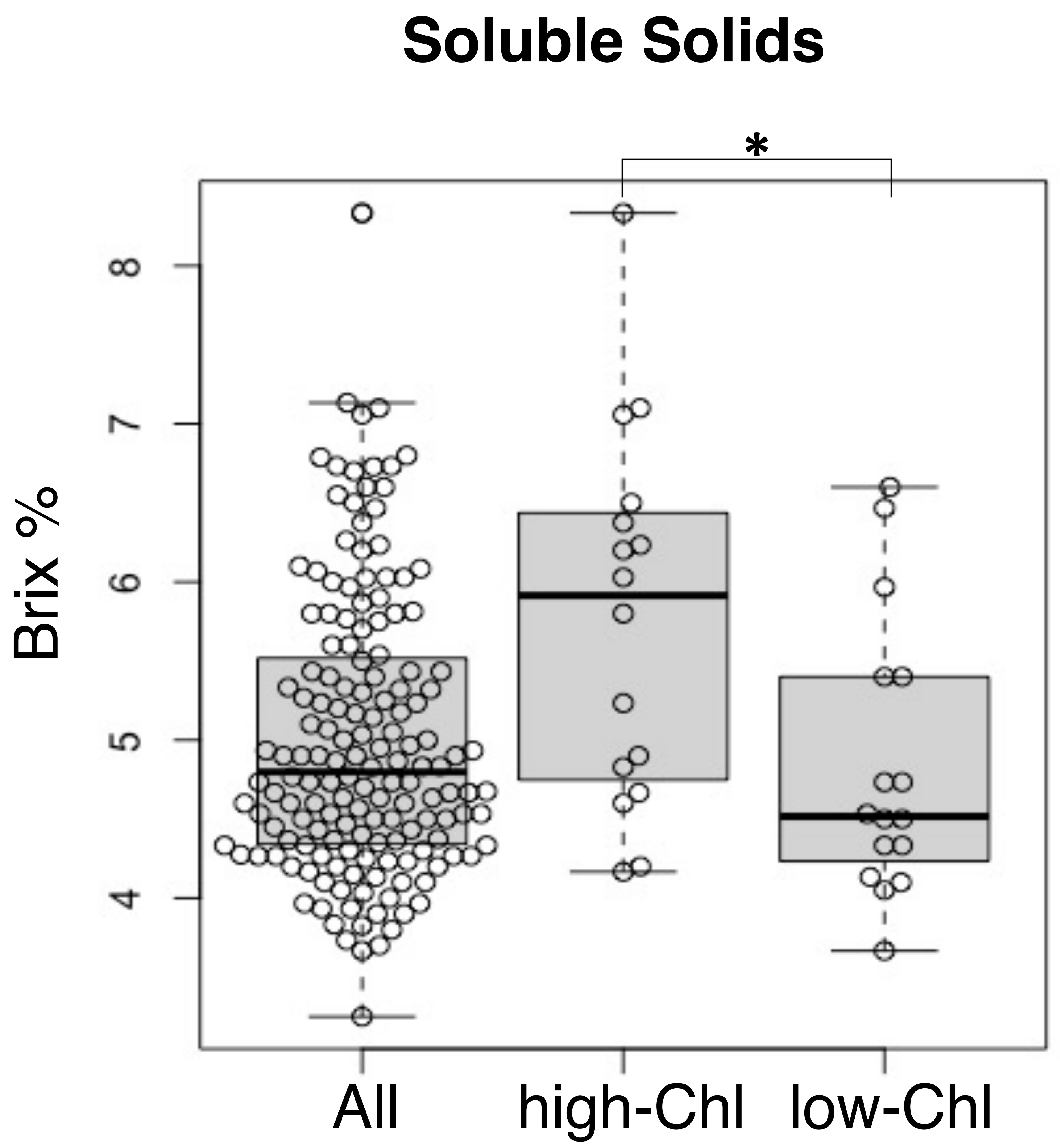

Figure S4. Comparison of soluble solids content in high and low chlorophyll varieties. Soluble solids content in fruits of the 156 varieties was obtained from data in Tieman et al (2017). Content in fruits of the 156 varieties (All), top 16 varieties with high chlorophyll a content (high-Chl) and bottom 16 varieties with low chlorophyll a content (low-Chl) are shown. Asterisk represents a significant difference (* $p < 0.05$) assayed by Welch's *t*-test.

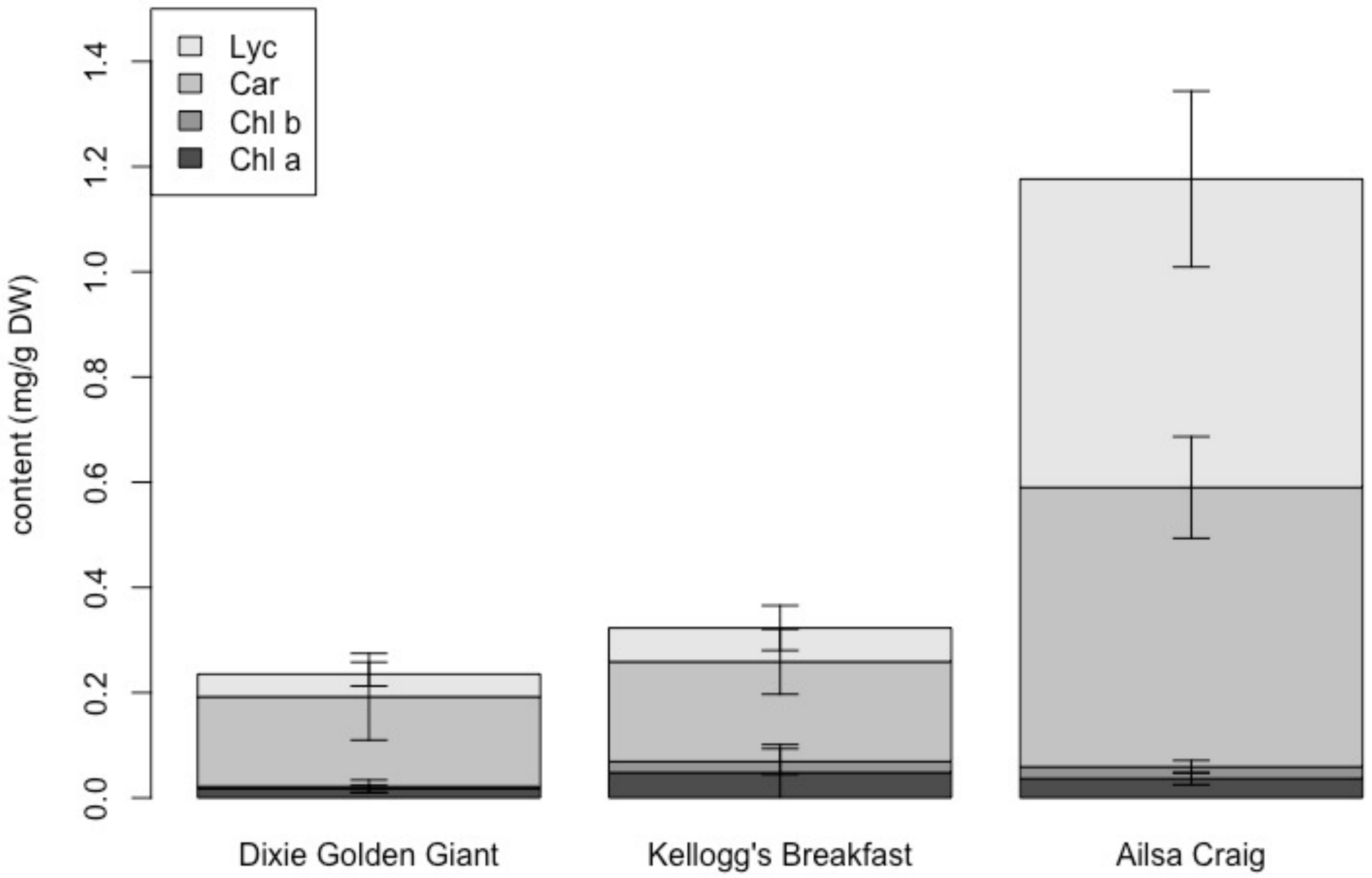

Figure S5. Comparison of amount of the four pigments in fruits of ‘Dixie Golden Giant’, ‘Kellogg’s Breakfast’, and ‘Ailsa Craig’. (a) Content of chlorophyll a (Chl a), chlorophyll b (Chl b), total carotenoids except for lycopene (Car), and lycopene (Lyc) are shown. The variety ‘Ailsa Craig’ was chosen as the control.

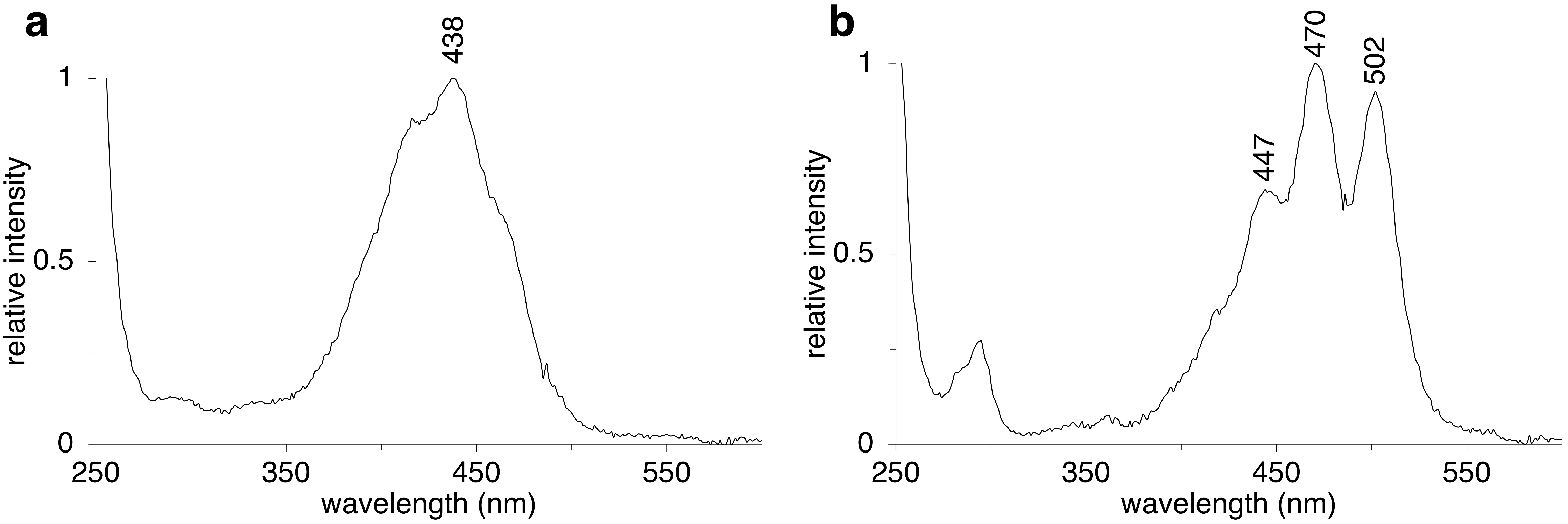

Figure S6. UV spectra of (**a**) peak #9 and (**b**) all-*trans*-lycopene. Values on each spectrum indicate wavelength at absorption maxima.
